# Pharmacokinetics, absolute bioavailability and tolerability of ketamine after intranasal administration to dexmedetomidine sedated dogs

**DOI:** 10.1371/journal.pone.0227762

**Published:** 2020-01-13

**Authors:** Lise Vlerick, Mathias Devreese, Kathelijne Peremans, Robrecht Dockx, Siska Croubels, Luc Duchateau, Ingeborgh Polis

**Affiliations:** 1 Small Animal Department, Faculty of Veterinary Medicine, Ghent University, Merelbeke, Belgium; 2 Department of Pharmacology, Toxicology and Biochemistry, Faculty of Veterinary Medicine, Ghent University, Merelbeke, Belgium; 3 Department of Veterinary Medical Imaging and Small Animal Orthopaedics, Faculty of Veterinary Medicine, Ghent University, Merelbeke, Belgium; 4 Department of Psychiatry and Medical Psychology, Ghent Experimental Psychiatry (GHEP) lab, Ghent University, Ghent, Belgium; 5 Biometrics Research Centre, Faculty of Veterinary Medicine, Ghent University, Merelbeke, Belgium; University of Bari, ITALY

## Abstract

Intranasal ketamine has recently gained interest in human medicine, not only for its sedative, anaesthetic or analgesic properties, but also in the management of treatment resistant depression, where it has been shown to be an effective, fast acting alternative treatment. Since several similarities are reported between human psychiatric disorders and canine anxiety disorders, intranasal ketamine could serve as an alternative treatment for anxiety disordered dogs. However, to the authors knowledge, intranasal administration of ketamine and its pharmacokinetics have never been described in dogs. Therefore, this study aimed to examine the pharmacokinetics, absolute bioavailability and tolerability of intranasal ketamine administration compared with intravenous administration. Seven healthy, adult laboratory Beagle dogs were included in this randomized crossover study. The dogs received 2 mg/kg body weight ketamine intravenously (IV) or intranasally (IN), with a two-week wash-out period. Prior to ketamine administration, dogs were sedated intramuscularly with dexmedetomidine. Venous blood samples were collected at fixed times until 480 min post-administration and ketamine plasma concentrations were determined by liquid chromatography-tandem mass spectrometry. Cardiovascular parameters and sedation scores were recorded at the same time points. Non-compartmental pharmacokinetic analysis revealed a rapid (Tmax = 0.25 ± 0.14 h) and complete IN bioavailability (F = 147.65 ± 49.97%). Elimination half-life was similar between both administration routes (T1/2el IV = 1.47 ± 0.24 h, T1/2el IN = 1.50 ± 0.97 h). Heart rate and sedation scores were significantly higher at 5 and 10 min following IV administration compared to IN administration, but not at the later time-points.

## Introduction

Ketamine is a dissociative anesthetic commonly used in veterinary medicine, mainly for induction and maintenance of anesthesia, but also for pain management in the peri- and postoperative period [1,2]. Generally ketamine is administered intravenously, intramuscularly or subcutaneously. Recently, intranasal administration of ketamine has gained attention in human medicine. Intranasal drug administration delivers drugs directly to the central nervous system, bypassing the blood brain barrier and is associated with a fast onset of action [3,4]. In addition, intranasal drug delivery avoids painful parenteral administration. Intranasal ketamine has been successfully used for sedation and premedication of pediatric patients [5–7] and in pain management of both children and adults [8–13]. Another application lies in psychiatry, where intranasal ketamine has been shown to be safe and effective for the treatment of major depressive disorders [14–16]. Moreover, recently, the US Food and Drug Administration approved S-ketamine nasal spray as an new therapy for treatment resistant depression. Since anxiety disorders in dogs show several similarities with human mood disorders [17–23], intranasal ketamine could also be a valuable alternative treatment for certain canine behavioural disorders. Brain imaging studies have reported similar abnormalities in regional cerebral blood flow of certain brain regions in dogs with pathological anxiety and in humans suffering from depression and anxiety disorders [17–23]. Furthermore, altered perfusion of these brain regions following intravenous subanesthetic ketamine administration has been demonstrated both in humans and dogs [24–30]. Additionally, functional imaging studies have demonstrated altered function of the serotonergic system in several cortical brain regions, both in humans and dogs suffering from mood and anxiety disorders [31–33]. Since the management of behavioural disorders in dogs is challenging and treatment outcome is often unsatisfactory, there is a need for faster and more effective treatment strategies [34–37]. Therefore, intranasal ketamine could be a promising adjunctive treatment in the management of canine anxiety disorders, complementary to standard behavioural therapy and pharmacotherapy. The intranasal route could also offer opportunities when ketamine is used for its anesthetic, sedative or analgesic properties in small animals. For example, when intravenous catheter placement is difficult due to medical or behavioural issues or for the ambulatory treatment of chronic pain. In cats, it has been demonstrated that intranasal administration of ketamine-midazolam is effective to induce sedation [38]. Moreover, compared with intramuscular administration of the same ketamine-midazolam combination, there were no differences in the measured parameters associated with sedation except for time to sternal recumbency, which was more rapid in the intranasal group. In rabbits, intranasal ketamine and S-ketamine was successfully used in combination with medetomidine to induce anesthesia [39]. Induction was more rapid compared to rabbits receiving medetomidine-ketamine intramuscularly or subcutaneously [40]. As rabbits are easily stressed, rapid onset of induction is desirable in this species. Intranasal ketamine has also been used in combination with dexmedetomidine and morphine to provide deep sedation sufficient for routine clinical examinations in rabbits [41]. However, to the authors knowledge, intranasal ketamine has never been used in dogs and its pharmacokinetics have never been described. Therefore, the primary objective of the present study was to determine and compare plasma concentrations of ketamine after intravenous and intranasal administration of 2 mg/kg body weight (BW) ketamine in healthy dogs. Secondly, the tolerability of a single intranasal ketamine administration was assessed.

## 1. Materials and methods

### 1.1. Animals

Seven neutered adult laboratory Beagles (5 females, 2 males; age 3.6 ± 1.7 years; weight 12.0 ± 2.6 kg) were included in a randomized crossover study. The animals were classified as healthy based on general clinical examination. Experiments were approved by the local Ethical Committee of the Faculty of Veterinary Medicine and of Bioscience Engineering, Ghent University (EC 2018_03) and all manipulations were performed according to good animal practice. Welfare of the animals was respected at each time and great care was taken to avoid stress and anxiety. No animals were sacrificed. The dogs were provided by the Small Animal Department of the Faculty of Veterinary Medicine and were purchased from Marshall BioResources (North Rose, New York, United States). The dogs were socially-housed in small groups (2 to 8 dogs), according to the European and Belgian legislation and received environmental enrichment (Directive 2010/63/EU, KB 29/05/2013). The bedding material in the inner part of the housing facility consisted of wood shavings. The dogs had permanent access to an outside area of 15 m^2^ and twice a day, they were allowed to run and play outside in an enclosed play area, enriched with climbing platforms, hiding places and small bushes. In addition, the dogs were regularly walked by students of the Faculty of Veterinary Medicine. Food was withheld for at least 12 hours before the start of the experiments, but water was provided ad libitum.

### 1.2. Study design

The dogs were randomly allocated to a two-period crossover design by the principal investigator, using an online randomization program (www.randomizer.org). Two routes of administration of ketamine were examined: the intravenous (IV) and the intranasal route (IN). Following a two-week wash-out period, each dog underwent the same protocol but receiving ketamine through a different administration route. Prior to each ketamine administration, the dogs were sedated intramuscular with dexmedetomidine (375 μg/m^2^ body surface, Dexdomitor^®^, Orion Corporation, Espoo, Finland). Following the placement of an IV 22G over-the-needle catheter (Optiva^®^, Jelco Smiths Medical International Ltd, Rossendale, UK) in one of the cephalic veins, the dogs were allowed to relax in a quiet room. The first blood sample (T0) was taken 20 minutes after dexmedetomidine injection and was followed by the administration of ketamine.

### 1.3. Drug administration and sample collection

A commercially available racemic ketamine preparation (Nimatek^®^, Eurovet Animal Health B.V., Bladel, the Netherlands) was used for IV injection. IV ketamine was administered at a dose of 2 mg/kg BW through the cephalic catheter, which was flushed with a standardized volume of saline prior to and following injection. For the nasal spray, an aqueous solution containing 2 mg/kg BW racemic ketamine (Nimatek^®^, Eurovet Animal Health B.V.) dissolved in 0.9% NaCl was administered to the mucous membranes of the nose using a mucosal atomization device (MAD Nasal^TM^, Wolfe Tory Medical, South Salt Lake City, Utah, United States). The total volume was fixed at 0.5 mL and was divided over the two nostrils. The MAD converted the aqueous solution into a fine mist creating a film coating the nasal mucosa. During the nasal administration, dogs were held in sternal recumbency with the head and neck gently dorsoflexed and were kept in this position for approximately 1 min after nasal delivery. Blood samples (each 2 mL) were collected from the vena jugularis before ketamine administration (T0) and at 2, 5, 10, 20, 30, 60, 120, 240, 360 and 480 min after IV ketamine administration. Following IN administration of ketamine, blood samples were taken at 5, 10, 20, 30, 45, 60, 120, 240, 360 and 480 min after dosing. Blood samples were immediately transferred into tubes containing lithium heparin and separated by centrifugation within 2 h at 3,500 rpm for 10 min. The plasma was harvested and stored at -80°C until analysis.

### 1.4. Tolerability assessment

Adverse reactions during and after intranasal drug administration were recorded to assess the tolerability (sneezing, coughing, head shaking, snorting and licking).

Sedation scores were determined at the same time points of blood collection. The degree of sedation was assessed with a modified numeric rating scale ranging from 0 (no sedation) to 15 (maximum sedation) [42] (Table 1).

**Table 1:** Numeric sedation rating scale, adapted from Gurney et al. (2009).

Parameter	Behaviour of the dog	Score
Spontaneous posture	Standing	0
	Sternally recumbent	1
	Laterally recumbent	2
Palpebral reflex	Brisk	0
	Slow	1
	Absent	2
Eye position	Forward (normal position)	0
	Rotated ventrally	2
Response to sound (handclap)	Body movement	0
	Head movement	1
	Ear twitch	2
	No reaction	3
Resistance to lateral recumbency	Full (stands)	0
	Moderate restraint required	1
	Mild restraint required	2
	No resistance	3
Overall appearance	No sedation apparent	0
	Mild sedation	1
	Moderate sedation	2
	Well sedated	3

Together with the scoring of sedation, the following physiological variables were monitored: heart rate, obtained by auscultation, respiratory rate, obtained by direct observation of thoracic excursions and systolic blood pressure, using an ultrasonic Doppler flow detector and an inflatable cuff.

### 1.5. Quantification of ketamine in plasma

Sample preparation was done as described by Devreese et al. (2015) [43]. The chromatographic system consisted of a Waters Alliance 2690 separation module and autosampler of the same type (Waters, Zellik, Belgium). Chromatographic separation was achieved on an Acquity UPLC^®^ BEH C18 column (particle diameter: 1.7 μm) (Waters). The mobile phases were (A) 0.1% formic acid in UPLC water and (B) 0.1% formic acid in methanol. The following gradient elution program was run: 0–1.0 min (95% A, 5% B), 1.0–2.0 min (linear gradient to 5% A), 2.0–5.5 min (5% A, 95% B), 5.5–6.0 (linear gradient to 95% A), 6.0–10.0 min (95% A, 5% B). Flow rate was set at 300 μL/min.

The LC column effluent was interfaced to a Waters Quattro Premier triple quadrupole mass spectrometer equipped with a heated electrospray ionization (h-ESI) probe operating in the positive ionization mode (Micromass Waters, UK). Acquisition was performed in the selected reaction monitoring (SRM) mode. The two most intense product ions of the precursor ion were monitored in the SRM mode for quantification and identification, respectively. For ketamine *m/z* 238.4 > 125.1/179.2 and for the internal standard (d3-ketamine) *m/z* 241.4 > 125.1/179.2. The method was validated according to a protocol previously described by [43]. The limit of quantification (LOQ) was 20 ng/mL and the linear range was 20–10,000 ng/mL.

### 1.6. Pharmacokinetic analysis

Plasma concentration-time profiles were modelled by non-compartmental analysis (NCA) using Phoenix 8.4 (Certara, NJ, USA). Plasma concentrations measured at 360 and 480 min after ketamine administration fell below the LOQ and were therefore not taken into account for pharmacokinetic analysis. The following major pharmacokinetic parameters were calculated: AUC_0-4h_ area under the plasma concentration-time curve from 0 to 4 hours post-administration; AUC_0-∞_ area under the plasma concentration-time curve from 0 to infinity; C_max_ maximal plasma concentration (IN); C_0_ plasma concentration at time zero (IV); T_max_ time to maximal plasma concentration (IN); Vd volume of distribution; Cl total body clearance; T_1/2el_ terminal elimination half-life; k_el_ elimination rate constant. Vd and Cl values after IN administration were not corrected for IN bioavailability (F). The absolute IN F, expressed as percentage, was calculated according to the following formula:

$$F=AUC_{0‐inf}IN/AUC_{0‐inf}IV\;*\;100.$$

### 1.7. Statistical analysis

A Student t-test was used to compare both administration routes (IV and IN) for each pharmacokinetic parameter. The level of significance was set at 0.05. For the evaluation of heart rate, respiratory rate, systolic blood pressure and sedation scores, linear mixed models were fitted onto the data set using dog as a random effect and time, treatment and their interaction as fixed effects as the data could be assumed to be normally distributed according to the Shapiro-Wilks test. Next, the two administration routes were compared at seven different time points (5, 10, 20, 30, 60, 120 and 240 min after ketamine administration) and tested at the Bonferroni adjusted significance level of 0.05/7 = 0.0071. Measurements at 360 and 480 min were not included in the analysis since plasma concentrations at these time points were also not taken into account for pharmacokinetic analysis.

## 2. Results

**Fig 1:**
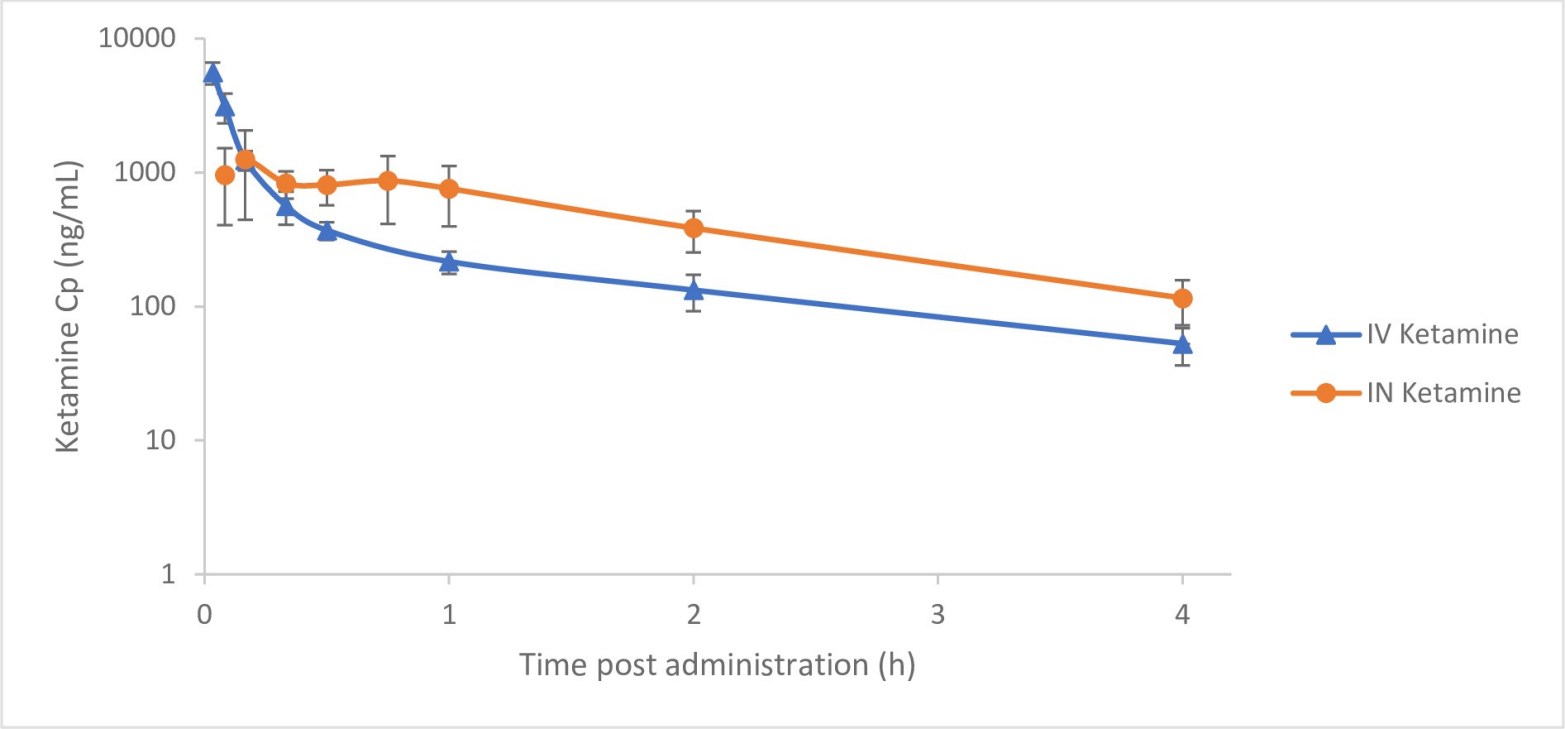
Mean plasma concentrations of ketamine in dogs administered a single dose of 2 mg/kg BW intravenously (IV) or intranasally (IN) (n = 7, mean ± SD).

Mean ketamine plasma concentration versus time curves for each administration route are displayed in Fig 1. Pharmacokinetic results are shown in Table 2. No significant differences in pharmacokinetic parameters were found between the two administration routes.

**Table 2:** Pharmacokinetic parameters of ketamine in dogs administered a single dose of 2 mg/kg BW intravenously (IV) or intranasally (IN) (n = 7, mean ± SD).

Pharmacokinetic parameter	IV	IN
AUC_0-4h_ (ng.h/mL)	1415.78 ± 338.40	1925.66 ± 789.83
AUC_0-∞_ (ng.h/mL)	1532.31 ± 397.01	2199.27 ± 773.23
C_max_ (ng/mL)	/	1694.48 ± 876.67
C_0_ (ng/mL)	9725.69 ± 3121.04	/
T_max_ (h)	/	0.25 ± 0.14
Vd (mL/kg)	2869.08 ± 570.03	2342.36 ± 2086.60
Cl (mL/h/kg)	1378.09 ± 340.02	1008.68 ± 333.27
T_1/2el_ (h)	1.47 ± 0.24	1.50 ± 0.97
k_el_ (1/h)	0.48 ± 0.07	0.61 ± 0.29
F (%)	100	147.65 ± 49.97

AUC_0-4h_ area under the plasma concentration-time curve from 0 to 4 hours post-administration; AUC_0-∞_ area under the plasma concentration-time curve from 0 to infinity; C_max_ maximal plasma concentration (IN); C_0_ plasma concentration at time zero (IV); T_max_ time to maximal plasma concentration (IN); Vd volume of distribution; Cl total body clearance; T_1/2el_ terminal elimination half-life; k_el_ elimination rate constant. Vd and Cl values after IN administration were not corrected for IN bioavailability (F).

Concerning the heart rate, significant differences between the two administration routes were found at two time points: 5 (*p* <0.001) and 10 min (*p* <0.001) after ketamine administration, with a higher heart rate following IV administration. At the same time points, significant differences in sedation scores were also observed (*p* <0.001) with higher sedation scores following IV administration compared to IN administration. No significant differences between the two administration routes were found for the respiratory rate and systolic blood pressure.

Dogs tolerated the IN administration relatively well. IN administration led to some degree of nasal irritation, reflected by sneezing, coughing and head shaking in almost all of the dogs (Table 3). However, these reactions were mild and only occurred immediately after nasal administration and disappeared fast.

**Table 3:** Adverse reactions following intranasal administration of 2 mg/kg BW racemic ketamine in the individual dogs (n = 7).

*Dog*	*Sneezing*	*Coughing*	*Head shaking*	*Snorting*	*Licking*	*Duration*
*Dog 1*	-	+	-	-	-	< 10 sec
*Dog 2*	+	-	+	+	-	< 1 min
*Dog 3*	+	+	-	+	+	< 1 min
*Dog 4*	-	+	-	-	-	< 1 min
*Dog 5*	+	-	-	+	-	< 10 sec
*Dog 6*	+	-	-	+	-	< 10 sec
*Dog 7*	-	-	-	-	-	-

## 3. Discussion

To our knowledge, this is the first study describing IN administration of ketamine in dogs and its pharmacokinetics and tolerance. Ketamine was rapidly absorbed after IN administration, with maximal plasma concentrations at 15 min and as early as 5 min in one subject. IN drug delivery is known to be associated with rapid drug absorption from the rich, IN vascular bed, leading to rapidly attained peak blood levels, as observed in this study [3]. Ketamine was completely bioavailable following IN administration. This is much higher than the reported IN bioavailability of 45 and 50% in human studies [44,45]. This can be partially explained by differences in anatomy of the nasal cavity between humans and dogs. Following intranasal administration, ketamine can be delivered directly to the central nervous system, where it can exert its actions [3,4,46]. This direct nose to brain delivery, bypassing the blood brain barrier, occurs mainly through the olfactory epithelium, which comprises 77% of the nasal cavity in dogs [46]. The human olfactory epithelium, on the contrary, is restricted to a small area in the roof of the nasal cavity and only covers less than 10% of the nasal cavity. The complete IN bioavailability can further be explained by the fact that the dogs were sedated, which facilitated nasal drug delivery and is associated with less risk of spilling and swallowing. Moreover, using a spray device instead of droplet administration of the drug into the nose and dividing the dose over the two nostrils would also enhance nasal absorption and bioavailability, since this increases the area over which the drug is spread [7,46].

Total body clearance (Cl) of ketamine was lower compared to other veterinary studies examining the pharmacokinetics of ketamine in dogs [47–49]. In the study of Pypendop and Ilkiw (2005), mean Cl after IV administration of ketamine was 3492 ± 1038 mL/h/kg, while volume of distribution at steady state (Vss) was 4060.3 ± 2405.7 mL/kg. Romagnoli et al. (2017) reported separate Cl values for S- (4309.2 ± 768 mL/h/kg) and R-ketamine (4048.8 ± 640.8 mL/h/kg) following IV racemic ketamine administration, with a volume of distribution for the central compartment of 750 ± 370 mL/kg. The study of Sandbaumhüter et al. consisted of two groups of dogs receiving ketamine, with one group anesthetized with sevoflurane and one group sedated with medetomidine. In the sevoflurane group, Cl was 3341.4 ± 664.2 mL/h/kg for R-ketamine and 3490.2 ± 642 mL/h/kg for S-ketamine with Vss of 1630 ± 1170 mL/kg and 1620 ± 1230 mL/kg respectively. In the medetomidine group Cl was 2844.6 ± 474 mL/h/kg for R-ketamine and 2745.6 ± 996.6 mL/h/kg for S-ketamine, with Vss of 3370 ± 660 mL/kg and 3130 ± 660 mL/kg respectively. Elimination half-life was comparable with that reported in the study of Pypendop and Ilkiw (2005) (1.57 ± 0.61 h), but was longer than documented by Romagnoli et al. (2017) (0.26 ± 0.12 h for S-ketamine and 0.26 ± 0.09 h for R-ketamine) and Sandbaumhüter et al. (2016) (0.50 ± 0.04 h for R-ketamine and 0.48 ± 0.42 h for S-ketamine in the sevoflurane group; 1.14 ± 0.17 h for R-ketamine and 1.11 ± 0.21 h for S-ketamine in the medetomidine group). The longer half-life of ketamine in the medetomidine group compared with the sevoflurane group in the study of Sandbaumhütter et al. (2017) indicates a slower elimination in the latter group. The lower clearance and subsequent longer half-life in the current study compared to the literature is in agreement with this and could be explained by the fact that the dogs were sedated with dexmedetomidine. Dexmedetomidine and medetomidine are strong inhibitors of the N-demethylation of ketamine to norketamine by competition for the binding site on cytochrome P450 enzymes [48]. Since the formation of norketamine is a major pathway in the biotransformation and hence elimination of ketamine, inhibition of norketamine formation can explain the slower elimination of ketamine when combined with dexmedetomidine. Moreover, since ketamine is a drug with a high hepatic extraction ratio in the dog [50], another explanation for the observed lower clearance and longer half-life could be the decreased cardiac output and reduced hepatic blood flow associated with dexmedetomidine administration [51,52]. In agreement with this, dexmedetomidine has also been shown to alter thiopental pharmacokinetics, most probably by decreasing cardiac output and regional blood flow [53].

Few data are available on ketamine’s effective analgesic serum concentrations. A study of Bergadano et al. (2009) [54], investigating the analgesic effects of a low-dose constant rate infusion of ketamine, showed that ketamine’s antinociceptive effects were evident when plasma concentrations ranged between 220–370 ng/mL. Another study investigating antinociceptive effects of different ketamine infusion regimes found that the serum concentration of ketamine to produce mechanical antinociceptive effects is above 200 ng/mL [55]. In the present study, mean Cmax after IN administration was 1694.48 ng/mL and mean plasma concentrations stayed above 200 ng/mL until 2 h after ketamine administration, indicating antinociceptive effects in this time frame. This implies that a single IN administration of 2 mg/kg BW ketamine has relatively long lasting analgesic effects in dogs. IN ketamine could therefore serve as a valuable alternative when IV catheter placement is difficult. However, these findings should be interpreted with caution, since this study was not designed to assess the analgesic or sedative potential of IN ketamine. Further studies evaluating these clinical effects of IN administered ketamine at different doses are necessary.

In both groups heart rate was low before ketamine administration, due to the sedation with dexmedetomidine (47 ± 5 beats per min (bpm) in the IV group and 43 ± 5 bpm in the IN group). It is known that dexmedetomidine causes bradycardia, with normal expected heart rates ranging between 45 and 60 bpm [51], which is in agreement with our observations. Following ketamine administration, heart rate increases in both groups, due to ketamine’s sympathomimetic effects [56]. However, the stimulatory effects of ketamine on the cardiovascular system were attenuated by the dexmedetomidine comedication. Heart rate and sedation scores were higher following IV administration compared to IN administration the first 10 min after ketamine administration. Following IV ketamine administration, heart rate increased to 86 ± 22 bpm, while after intranasal administration, only a small increase in heart rate was observed. These differences in heart rates and sedation scores can be explained by the fact that, during the first 10 min following ketamine administration, plasma concentrations were higher following IV administration compared to IN administration.

The seven dogs completed the study without clinical significant or serious adverse events. The IN administration was easy to perform and generally well tolerated by the sedated dogs, with short duration mild adverse effects associated with the route of administration. The most frequently observed adverse event in this study was a short sneezing and snorting reaction, which was present in 4 out of 7 dogs. A brief episode of sneezing during or after intranasal administration was also reported in dogs receiving intranasal midazolam [57]. In another study, examining nasal administration of diazepam in dogs, several dogs developed salivation and reverse sneezing following intranasal administration [58]. Also in cats, a sneezing and snorting reaction following intranasal administration of a ketamine-midazolam combination has been reported [38]. However, in the current study, coughing was also observed in 3 out of 7 dogs and head shaking was present in one dog. These reactions may be due to the bitter taste of ketamine, which could have caused irritation and an unpleasant sensation in the dogs. In human medicine, patients receiving intranasal ketamine frequently complain about a bad taste in the mouth, due to drug runoff into the pharynx [6,9,13,59].

A limitation of this study is the fact that the dogs were sedated with dexmedetomidine prior to the ketamine administration. Dexmedetomidine was administered to avoid behavioural and cardiovascular adverse effects due to the ketamine administration. Dexmedetomidine-ketamine is a frequently used combination in veterinary medicine as the two drugs are complementary to each other and side effects of both drugs are minimized when used together. Dexmedetomidine may attenuate or prevent tachycardia, hypertension, delirium, excitement and hallucinations caused by ketamine [51,60]. On the other hand, ketamine may reduce the bradycardia and hypotension associated with dexmedetomidine administration [61]. However, the use of co-medication can have an influence on drug disposition and pharmacokinetics.

## Conclusion

IN administration of 2 mg/kg BW ketamine to healthy dogs, sedated with dexmedetomidine, was well tolerated by all of the dogs. Rapid systemic absorption and complete bioavailability of IN ketamine were demonstrated. These findings encourage the use of IN ketamine in veterinary medicine, not only as anesthetic, sedative or analgesic but potentially also in the treatment of dogs with anxiety disorders. Further studies are necessary to assess the clinical effects of IN ketamine in anxiety-disordered dogs.

## Supporting information

S1 Table Heart rate (HR), respiratory rate (RR), systolic arterial blood pressure (SAP) and sedation score following intravenous (IV) and intranasal (IN) administration of 2 mg/kg BW racemic ketamine. **(n = 7, mean ± SD).** The degree of sedation was assessed with a modified numeric rating scale ranging from 0 (no sedation) to 15 (maximum sedation), adapted from Gurney et al. (2009).
(DOCX)

S2 Table Heart rate (HR), respiratory rate (RR), systolic arterial blood pressure (SAP) and sedation score (SS) at different time points (T) in the individual dogs following intravenous administration of 2 mg/kg BW racemic ketamine. T: min; HR: beats/min; RR: breaths/min; SAP: mm Hg; NA: not available. The degree of sedation was assessed with a modified numeric rating scale ranging from 0 (no sedation) to 15 (maximum sedation), adapted from Gurney et al. (2009).
(DOCX)

S3 Table Heart rate (HR), respiratory rate (RR), systolic arterial blood pressure (SAP) and sedation score (SS) at different time points (T) in the individual dogs following intranasal administration of 2 mg/kg BW racemic ketamine. T: min; HR: beats/min; RR: breaths/min; SAP: mm Hg; NA: not available. The degree of sedation was assessed with a modified numeric rating scale ranging from 0 (no sedation) to 15 (maximum sedation), adapted from Gurney et al. (2009).
(DOCX)
