# Supplementary material for: Pharmacokinetics, absolute bioavailability and tolerability of ketamine after intranasal administration to dexmedetomidine sedated dogs
Source: PLoS One. 2020 Jan 13;15(1):e0227762. doi: 10.1371/journal.pone.0227762 (PMC6957157; doi:10.1371/journal.pone.0227762)
Supplement: S1 Table — (n = 7, mean ± SD). The degree of sedation was assessed with a modified numeric rating scale ranging from 0 (no sedation) to 15 (maximum sedation), adapted from Gurney et al. (2009). (DOCX) [file pone.0227762.s001.docx]

**S1 Table: Heart rate (HR), respiratory rate (RR), systolic arterial blood pressure (SAP) and sedation score following intravenous (IV) and intranasal (IN) administration of 2 mg/kg BW racemic ketamine. (n=7, mean ± SD).**

|  | IV ketamine |  |  |  | IN ketamine |  |  |  |
| --- | --- | --- | --- | --- | --- | --- | --- | --- |
| Time (min) | HR (beats/min) | RR (breaths/min) | SAP (mm Hg) | Sedation score | HR (beats/min) | RR (breaths/min) | SAP (mm Hg) | Sedation score |
| 0 | 47 ± 5 | 16 ± 3 | 145 ±13 | 7 ± 4 | 43 ± 5 | 15 ± 5 | 130 ± 12 | 8 ± 2 |
| 2 | 86 ± 22 | 13 ± 4 | 159 ± 16 | 11 ± 0 | / | / | / | / |
| 5 | 80 ± 18 | 15 ± 5 | 163 ± 14 | 11 ± 0 | 49 ± 6 | 13 ± 6 | 139 ± 15 | 7 ± 2 |
| 10 | 70 ± 17 | 16 ± 9 | 163 ± 17 | 11 ± 0 | 50 ± 4 | 14 ± 4 | 146 ± 15 | 8 ± 2 |
| 20 | 59 ± 8 | 15 ± 5 | 146 ± 19 | 10 ± 1 | 52 ± 8 | 14 ± 8 | 139 ± 13 | 8 ± 2 |
| 30 | 55 ± 6 | 15 ± 5 | 139 ± 20 | 9 ± 2 | 50 ± 4 | 13 ± 5 | 136 ± 14 | 8 ± 2 |
| 45 | / | / | / | / | 50 ± 6 | 13 ± 7 | 133 ± 14 | 8 ± 2 |
| 60 | 53 ± 4 | 18 ± 6 | 136 ± 23 | 7 ± 3 | 49 ± 5 | 13 ± 6 | 131 ± 19 | 7 ± 2 |
| 120 | 51 ± 7 | 15 ± 4 | 138 ± 21 | 4 ±2 | 49 ± 5 | 13 ± 6 | 136 ± 15 | 5 ± 2 |
| 240 | 63 ± 10 | 18 ± 3 | 136 ± 18 | 0 ± 1 | 65 ± 14 | 15 ± 4 | 136 ± 9 | 1 ± 1 |

The degree of sedation was assessed with a modified numeric rating scale ranging from 0 (no sedation) to 15 (maximum sedation), adapted from Gurney et al. (2009).
