# Supplementary material for: Pharmacokinetics, absolute bioavailability and tolerability of ketamine after intranasal administration to dexmedetomidine sedated dogs
Source: PLoS One. 2020 Jan 13;15(1):e0227762. doi: 10.1371/journal.pone.0227762 (PMC6957157; doi:10.1371/journal.pone.0227762)
Supplement: S2 Table — T: min; HR: beats/min; RR: breaths/min; SAP: mm Hg; NA: not available. The degree of sedation was assessed with a modified numeric rating scale ranging from 0 (no sedation) to 15 (maximum sedation), adapted from Gurney et al. (2009). (DOCX) [file pone.0227762.s002.docx]

**S2 Table: Heart rate (HR), respiratory rate (RR), systolic arterial blood pressure (SAP) and sedation score (SS) at different time points (T) in the individual dogs following intravenous administration of 2 mg/kg BW racemic ketamine.**

|  | ***Dog 1*** | | | | ***Dog 2*** | | | | ***Dog 3*** | | | | ***Dog 4*** | | | | ***Dog 5*** | | | | ***Dog 6*** | | | | ***Dog 7*** | | | |
| --- | --- | --- | --- | --- | --- | --- | --- | --- | --- | --- | --- | --- | --- | --- | --- | --- | --- | --- | --- | --- | --- | --- | --- | --- | --- | --- | --- | --- |
| **T** | **HR** | **RR** | **SAP** | **SS** | **HR** | **RR** | **SAP** | **SS** | **HR** | **RR** | **SAP** | **SS** | **HR** | **RR** | **SAP** | **SS** | **HR** | **RR** | **SAP** | **SS** | **HR** | **RR** | **SAP** | **SS** | **HR** | **RR** | **SAP** | **SS** |
| 0 | NA | 16 | 135 | 3 | 48 | 16 | 150 | 7 | 40 | 20 | 160 | 9 | 48 | 16 | 145 | 4 | 52 | 20 | 160 | 4 | 52 | 12 | 125 | 13 | 42 | 12 | 140 | 11 |
| 2 | NA | 12 | 140 | NA | 80 | 12 | 180 | 11 | NA | 16 | 175 | 11 | 92 | 8 | 165 | 11 | 112 | 12 | 155 | 11 | 92 | 12 | 140 | 11 | 52 | 20 | 160 | 11 |
| 5 | 80 | 24 | 165 | NA | 72 | 16 | 165 | 11 | 120 | 16 | 190 | 11 | 72 | 8 | 165 | 11 | 72 | 12 | 145 | 11 | 80 | 12 | 150 | 11 | 64 | 20 | 160 | 11 |
| 10 | 60 | 20 | 160 | 10 | 64 | 20 | 165 | 11 | 100 | 16 | 180 | 11 | 60 | 12 | 155 | 10 | 64 | 16 | 170 | 11 | 56 | 16 | 130 | 11 | 88 | 12 | 180 | 11 |
| 20 | 52 | 20 | 140 | 10 | 52 | 16 | 150 | 10 | 56 | 20 | 150 | 10 | 52 | 10 | 140 | 11 | 68 | 12 | 180 | 7 | 60 | 8 | 115 | 10 | 72 | 16 | 150 | 11 |
| 30 | 52 | 20 | 140 | 10 | 44 | 12 | 165 | 11 | 56 | 24 | 135 | 10 | 56 | 8 | 150 | 7 | 56 | 16 | 150 | 8 | 60 | 12 | 105 | 8 | 60 | 16 | 125 | 11 |
| 60 | 52 | 16 | 150 | 6 | 52 | 24 | 105 | 10 | 48 | 24 | 155 | 8 | 56 | 16 | 150 | 4 | 56 | 24 | 160 | 3 | 56 | 12 | 105 | 7 | 48 | 12 | 130 | 10 |
| 120 | 52 | 20 | 130 | 4 | 40 | 12 | 135 | 6 | 52 | 20 | 180 | 5 | 56 | 12 | 145 | 1 | 48 | 12 | 130 | 3 | 64 | 16 | 110 | 3 | 48 | 16 | 135 | 4 |
| 240 | 52 | 20 | 120 | 1 | 52 | 16 | 140 | 1 | 60 | 24 | 150 | 0 | 76 | 16 | 140 | 0 | 72 | 16 | 160 | 0 | 72 | 16 | 105 | 0 | 60 | 16 | 140 | 1 |

T: min; HR: beats/min; RR: breaths/min; SAP: mm Hg.; NA: not available. The degree of sedation was assessed with a modified numeric rating scale ranging from 0 (no sedation) to 15 (maximum sedation), adapted from Gurney et al. (2009).
