# Supplementary material for: Pharmacokinetics, absolute bioavailability and tolerability of ketamine after intranasal administration to dexmedetomidine sedated dogs
Source: PLoS One. 2020 Jan 13;15(1):e0227762. doi: 10.1371/journal.pone.0227762 (PMC6957157; doi:10.1371/journal.pone.0227762)
Supplement: S3 Table — T: min; HR: beats/min; RR: breaths/min; SAP: mm Hg; NA: not available. The degree of sedation was assessed with a modified numeric rating scale ranging from 0 (no sedation) to 15 (maximum sedation), adapted from Gurney et al. (2009). (DOCX) [file pone.0227762.s003.docx]

**S3 Table: Heart rate (HR), respiratory rate (RR), systolic arterial blood pressure (SAP) and sedation score (SS) at different time points (T) in the individual dogs following intranasal administration of 2 mg/kg BW racemic ketamine.**

|  | ***Dog 1*** | | | | ***Dog 2*** | | | | ***Dog 3*** | | | | ***Dog 4*** | | | | ***Dog 5*** | | | | ***Dog 6*** | | | | ***Dog 7*** | | | |
| --- | --- | --- | --- | --- | --- | --- | --- | --- | --- | --- | --- | --- | --- | --- | --- | --- | --- | --- | --- | --- | --- | --- | --- | --- | --- | --- | --- | --- |
| **T** | **HR** | **RR** | **SAP** | **SS** | **HR** | **RR** | **SAP** | **SS** | **HR** | **RR** | **SAP** | **SS** | **HR** | **RR** | **SAP** | **SS** | **HR** | **RR** | **SAP** | **SS** | **HR** | **RR** | **SAP** | **SS** | **HR** | **RR** | **SAP** | **SS** |
| 0 | 44 | 20 | 130 | 8 | 40 | 20 | 145 | 9 | 36 | 20 | 140 | 10 | 40 | 8 | 120 | 6 | 44 | 12 | 135 | 6 | 52 | 12 | 110 | 7 | 48 | 16 | 130 | 10 |
| 5 | 40 | 12 | 140 | 5 | 48 | 24 | 130 | 5 | 48 | 16 | 165 | 10 | 48 | 6 | 125 | 6 | 60 | 8 | 125 | 6 | 48 | 12 | 150 | 8 | NA | 12 | 140 | 11 |
| 10 | 52 | 16 | 150 | 6 | 52 | 16 | 160 | 7 | 52 | 20 | 170 | 10 | 48 | 8 | 135 | 6 | 48 | 12 | 140 | 8 | 56 | 12 | 130 | 7 | 44 | 12 | 135 | 11 |
| 20 | 48 | 16 | 125 | 6 | 44 | 20 | 160 | 6 | 60 | 28 | 140 | 11 | 52 | 4 | 130 | 7 | 64 | 8 | 140 | 8 | 44 | 12 | 125 | 7 | 52 | 12 | 150 | 11 |
| 30 | 48 | 12 | 120 | 6 | 44 | 12 | 150 | 9 | 48 | 24 | 155 | 10 | 52 | 8 | 135 | 6 | 56 | 12 | 125 | 9 | 52 | 12 | 125 | 8 | 48 | 8 | 145 | 11 |
| 45 | 52 | 9 | 140 | 5 | 52 | 20 | 140 | 9 | 40 | 24 | 145 | 10 | 44 | 5 | 120 | 7 | 60 | 12 | 120 | 8 | 48 | 8 | 115 | 8 | 52 | 12 | 150 | 8 |
| 60 | 44 | 12 | 125 | 5 | 48 | 20 | 160 | 10 | 44 | 20 | 155 | 10 | 52 | 5 | 120 | 6 | 56 | 12 | 110 | 6 | 52 | 8 | 125 | 7 | 44 | 12 | 125 | 8 |
| 120 | 44 | 12 | 120 | 3 | 52 | 20 | 160 | 7 | 44 | 20 | 145 | 7 | 56 | 12 | 120 | 3 | 52 | 16 | 140 | 4 | 52 | 8 | 145 | 4 | 44 | 16 | 125 | 4 |
| 240 | 68 | 16 | 120 | 0 | 60 | 20 | 140 | 1 | 48 | 20 | 140 | 2 | 92 | 12 | 145 | 0 | 64 | 12 | 125 | 0 | 68 | 12 | 140 | 0 | 56 | 12 | 140 | 1 |

T: min; HR: beats/min; RR: breaths/min; SAP: mm Hg, NA: not available. The degree of sedation was assessed with a modified numeric rating scale ranging from 0 (no sedation) to 15 (maximum sedation), adapted from Gurney et al. (2009).
